# Development and Applications of a 1K SNP Panel for Whiteleg Shrimp: From Pedigree Reconstruction to Genomic Selection

**DOI:** 10.3390/ijms27114665

**Published:** 2026-05-22

**Authors:** Qiang Fu, Guangfeng Qiang, Ping Wang, Mianyu Liu, Kun Luo, Baolong Chen, Xianhong Meng, Xiang Zou, Ping Dai, Junyu Liu, Shiwei Zhang, Jie Kong, Sheng Luan

**Affiliations:** 1 State Key Laboratory of Mariculture Biobreeding and Sustainable Goods, Yellow Sea Fisheries Research Institute, Chinese Academy of Fishery Sciences, Qingdao 266071, China; fuqiang@ysfri.ac.cn (Q.F.);; 2 Laboratory for Marine Fisheries Science and Food Production Processes, Qingdao Marine Science and Technology Center, Qingdao 266237, China; 3 Sanya Marine Research Institute, Ocean University of China, Sanya 572000, China

**Keywords:** *Litopenaeus vannamei*, SNP array, family selection, pedigree reconstruction, breeding value estimation

## Abstract

*Litopenaeus vannamei*, the most widely farmed crustacean, relies on family-based selection where accurate pedigree information is essential. Although SNP-based tools offer high-accuracy pedigree assignment, adoption in commercial breeding remains limited. In this study, we developed a commercially viable 1K SNP panel with 1125 markers. Markers were selected from a 55K SNP dataset comprising 2330 individuals. We established a practical pedigree reconstruction workflow and implemented the panel in a field breeding population. The population included a selection group where families were reared separately and a test group where individuals were communally reared. We introduced anchor individuals from the selection group to enable pedigree linkage. All 1818 individuals from 72 families were accurately assigned. Family reconstruction achieved 100% consistency with known records, even when parents were partially missing. Heritability estimates for harvest weight ranged from 0.32 to 0.36 using pedigree-based BLUP (PBLUP), genomic BLUP (GBLUP), and single-step genomic BLUP (ssGBLUP). The ssGBLUP model, using a 0.15 to 0.85 weighting of G and A, achieved 6.67% and 19.40% higher accuracy than PBLUP and GBLUP. The panel also supported population structure analysis and diversity monitoring, demonstrating its value for genomic evaluation in commercial *L. vannamei* breeding.

## 1. Introduction

Whiteleg shrimp (*Litopenaeus vannamei*) is the most widely produced crustacean and plays a pivotal role in global shrimp production. In 2022, its total output reached 6.83 million tons according to FAO, making it the largest aquaculture species by volume. As the industry continues to expand, selective breeding programs have become essential for improving economically important traits, such as growth and disease resistance [1,2,3], which are vital for ensuring the long-term sustainability and profitability of shrimp farming.

Family-based selection is the predominant approach in shrimp genetic improvement programs, typically conducted within a nucleus population (NP) composed of selection and test groups [4]. To ensure biosecurity and minimize cross-contamination risks, full-sib families in the selection group are reared in separate tanks. Performance testing is then conducted in the test group, where individuals from different families are reared communally to minimize environmental variance. Under such communal rearing conditions, accurate pedigree information is essential for predicting estimated breeding values (EBVs), optimizing mating strategies, and controlling inbreeding [5,6,7]. However, traditional physical tagging methods, such as visible implant fluorescent elastomer (VIE) [8,9], have several limitations, including labor-intensive procedures [10], tagging errors [11,12], and the need for extended rearing to reach tagging size [13]. Shrimp typically require an additional 40–60 days to exceed 2 g for tagging, during which unequal environmental conditions and an increased risk of handling errors may introduce environmental bias and compromise family identity, thereby reducing the accuracy of family performance evaluations [14,15,16].

Single-Nucleotide Polymorphisms (SNPs) offer a promising alternative for pedigree reconstruction in aquaculture breeding programs. SNP-based parentage and sibship assignment provide higher accuracy, allow early communal rearing of families, and overcome the limitations of physical tagging. Beyond pedigree reconstruction, SNPs facilitate additional applications, including the estimation of genetic parameters, the prediction of genomic estimated breeding values (GEBVs), and the analysis of population structure and genetic diversity [17,18,19,20]. Furthermore, the development of low-density SNP panels has made high-throughput, cost-effective genotyping feasible, supporting broader adoption of genomic tools in large-scale commercial breeding operations [19,21]. These advantages make SNP technology a powerful tool for improving the accuracy and efficiency of selective breeding programs.

Although SNP-based pedigree reconstruction holds great promise for application in selective breeding programs, most studies on aquatic species have primarily focused on evaluating the minimum number of SNPs required to ensure parentage assignment accuracy under different mating and genetic diversity scenarios [22]. In general, approximately 100 high-quality SNPs were sufficient for reliable parentage assignment [23,24,25], while 300 to 500 markers might be required under more complex kinship structures [7,20]. In *L. vannamei*, low-density SNP panels have also been developed, ranging from 88 SNPs (Fluidigm SNPtype technology) [10] to 1169 SNPs (Target sequencing by PCR) [20], with their family identification accuracy assessed. Despite technological advances, the practical implementation of SNP-based methods as a complete replacement for VIE tagging remained rare in commercial shrimp breeding. This may be due to the lack of cost-effective SNP genotyping platforms that are accessible and scalable for routine use in large-scale breeding programs, as well as the absence of a well-defined operational framework that includes early-stage communal rearing tests, optimized sampling strategies, and standardized family identification protocols. Moreover, the broader potential of low-density SNP panels, for example, for estimating genomic genetic parameters and enhancing GEBV prediction accuracy, remained largely unexplored in practice.

This study aimed to develop and validate a cost-effective 1K SNP panel for *L. vannamei*, as part of the “Yellow Sea Array No. 1” system. The panel was specifically designed for accurate pedigree reconstruction of NP families that are communally reared from the early post-larval stage through to harvest. We also proposed a strategy using anchor full-sib individuals to address issues of missing parental samples. Beyond pedigree tracing, we evaluated the panel’s broader applications, including population structure analysis, genetic diversity monitoring, genetic parameter estimation, and GEBV prediction. To our knowledge, this was the first comprehensive and applied investigation of a low-density SNP panel in a commercial *L. vannamei* breeding population in China, offering a scalable molecular solution for practical family-based selective breeding programs in shrimp.

## 2. Results

### 2.1. Evaluation of the 1K SNP Panel Across Parent, Anchor and Test Groups

**Figure 1:**
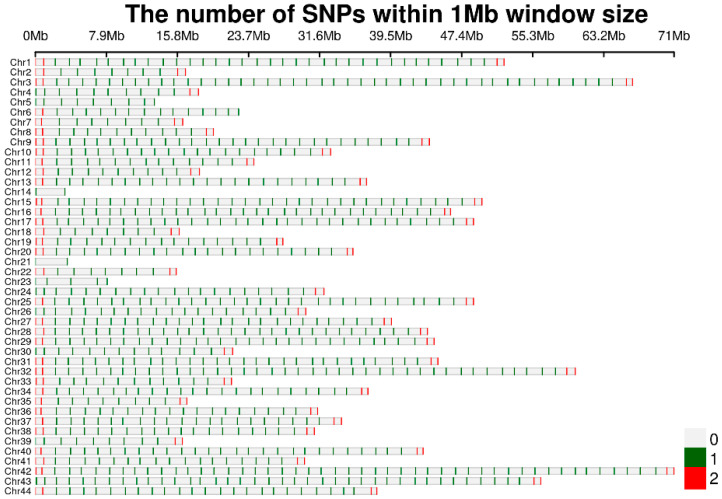
The distribution of 1125 SNPs in the 1K panel across the 44 chromosomes of *L. vannamei*. Color legend: White represents 0 SNPs, green represents 1 SNP, and red represents 2 SNPs in the 1Mb window.

**Figure 2:**
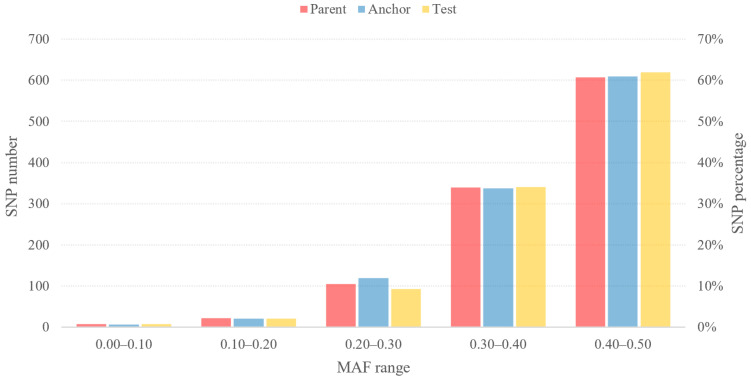
SNP number and percentage from 1K panel across MAF intervals in three groups.

The 1K SNP panel loci were evenly distributed across all 44 linkage groups as designed (Figure 1), providing genome-wide coverage. After quality control, 1081, 1092, and 1082 SNPs were retained in the parent, anchor, and test groups, respectively. MAF ranged from 0.05 to 0.50, with average MAF values of 0.40, 0.39 and 0.40 in three groups, and a mean PIC value of 0.36 (Table 1). More than 87% of the loci had an MAF greater than 0.30, while over 56% exceeded 0.40 (Figure 2), demonstrating the panel’s high polymorphism and informativeness. These results confirm the effectiveness of the marker selection strategy, which prioritized high MAF, moderate heterozygosity, and reliable call rates. Since all three groups originated from the same breeding population, their nearly identical MAF distributions across groups confirm the expected genetic consistency.

### 2.2. Population Genetic and Genetic Diversity Analysis

**Figure 3:**
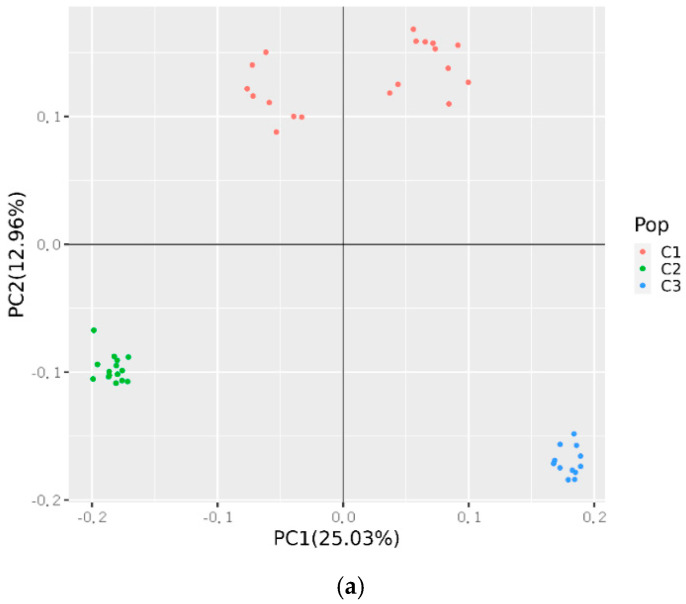

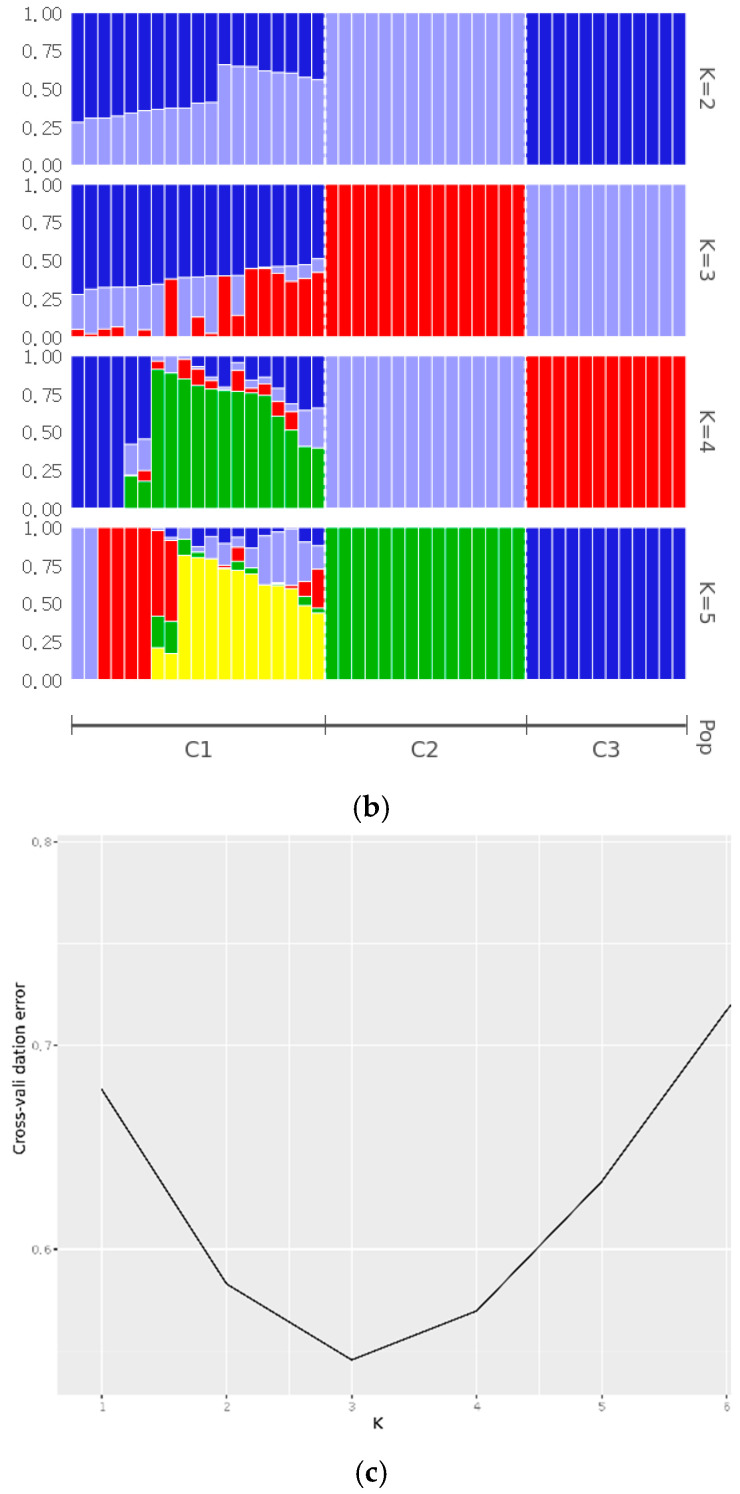
Population structure of three founder populations based on “Yellow Sea Array No.1” 1K SNP panel. (**a**) Principal component analysis of the founder individuals. Different colors represent different populations: C1, C2, and C3 correspond to different batches of introduced CP founder populations. (**b**) Population structure of the three founder populations for K = 2 to K = 5. Each individual is represented by a vertical line divided into K segments, where K is the number of assumed ancestral clusters; the colored segment shows the individual’s estimated membership proportions in a given cluster. (**c**) Graph showing the cross-validation error used to determine the optimal K value in the population structure analysis.

To assess the panel’s ability to resolve population structure, 46 founder individuals from three breeding populations were genotyped. Despite the limited sample size, PCA results showed clear separation among the three groups (Figure 3a), while Admixture analysis supported the optimal clustering at K = 3, corresponding to the lowest CV error (0.54) and consistent with their known origins (Figure 3b,c). These results demonstrated that the 1K SNP panel provides sufficient resolution for population genetic structure analysis, which may arise from stochastic drift or genetic differentiation. Genetic diversity was assessed in three groups: the founder populations (*n* = 46), the G5 test individuals (*n* = 1818), and the G5 parents (*n* = 117). Across all groups, the average MAF exceeded 0.39, and the mean PIC was above 0.35. The observed heterozygosity (Ho), expected heterozygosity (He), and nucleotide diversity (Pi) were also comparable across groups (Table 2). This indicated that the 1K panel effectively captures representative genome-wide diversity, even though the number of individuals used for evaluation varied greatly among populations. These results further confirm the panel’s suitability for both population structure and genetic diversity analysis.

### 2.3. Parentage Assignment and Sibship Analysis

**Figure 4:**
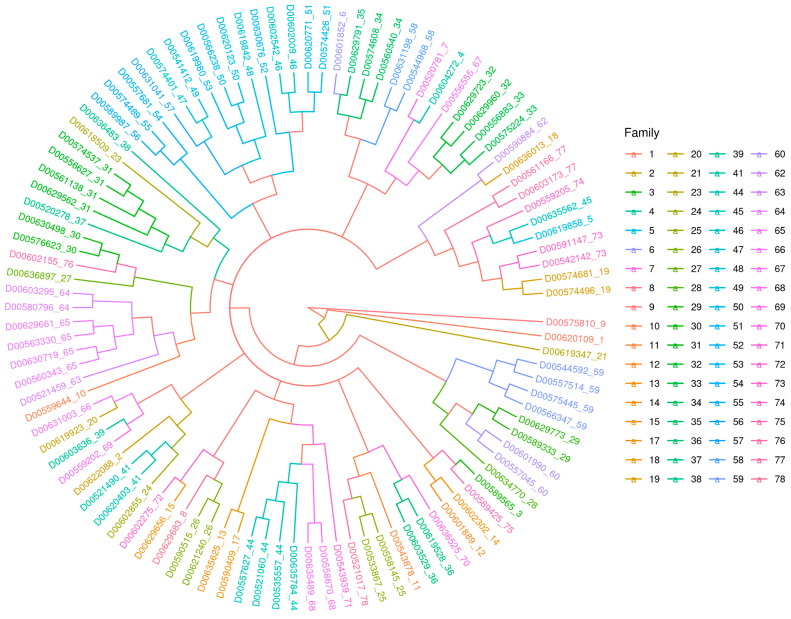
Phylogenetic tree of 103 anchor individuals from 72 families. Different colors represent individuals from different families (Bootstrap support values are shown in Supplementary Figure S3).

**Figure 5:**
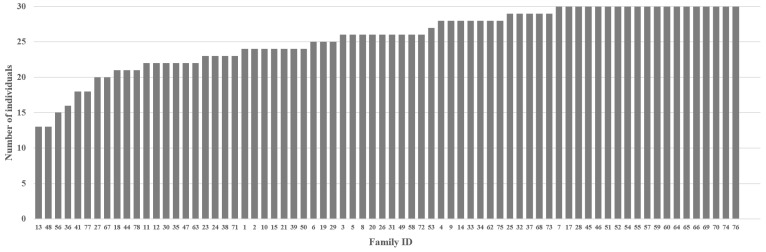
Number of test individuals in 72 families identified by Colony.

**Figure 6:**
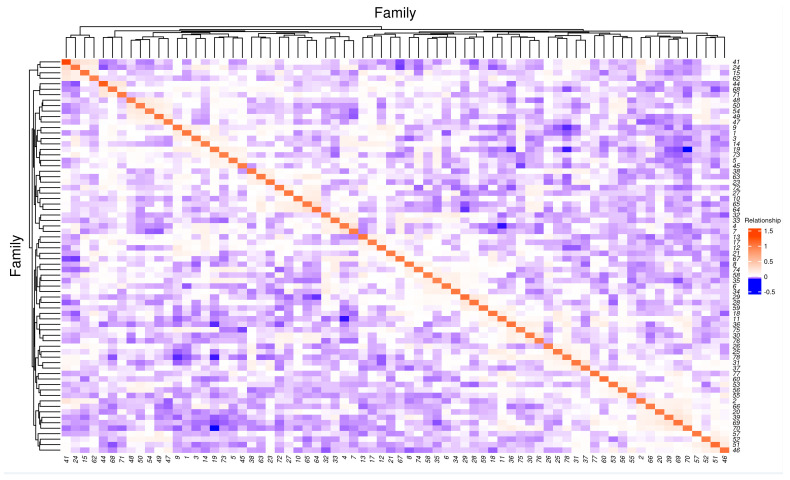
Kinship relationship matrix between pairs of full-sib families.

Using genotypes from 103 anchor individuals, COLONY successfully identified 72 full-sib families through sibship clustering. The inferred family structure was fully consistent with experimental records, and the phylogenetic tree of anchor individuals further confirmed that they were genetically distinguishable using the 1K panel (Figure 4). These results validate the reliability of anchor-based pedigree reconstruction. Genotypic data from 117 parental individuals were then incorporated to replace virtual parents with real ones. Parentage assignments were consistent with expected relationships, further confirming the accuracy of the reconstructed pedigree. Subsequently, all 1818 test individuals were assigned to the established families. Family sizes ranged from 13 to 30, with an average of 25.2 individuals per family (Figure 5), and no individuals remained unassigned. Additionally, the “Probability” output by the colony was 1.00 in all steps of the clustering analysis. Detailed information on parent–anchor relationships and offspring numbers of all families is provided in Supplementary Figure S1. Additionally, the pairwise kinship matrix heatmap for the 72 full-sib families (Figure 6) revealed kinship coefficients ranging from −0.10 to 0.29, with a mean of −0.01. Only a minimal number of pairwise kinship coefficients exceeded 0.1. These results demonstrated that kinship and inbreeding relationships among the families were effectively managed and controlled throughout the breeding program.

To evaluate the robustness of the method, a separate analysis was performed using only the genotypes of test individuals, without anchor or parental data. This analysis identified the same number of families with entirely consistent composition (although specific family IDs could not be determined in this case). Additionally, the phylogenetic tree further supported these results by confirming the classification of individuals into 72 groups (Supplementary Figure S2). These results demonstrate that the 1K SNP panel enables accurate sibship inference even in the absence of reference individuals.

### 2.4. Variance Components, Heritability Estimates and Prediction Accuracy

Table 3 summarizes the descriptive statistics of harvest body weight by sex among the 1818 test individuals. Overall, body weight showed considerable variation, with a coefficient of variation of 25.47%. Male shrimp had slightly lower average body weight and standard deviation compared to females. Table 4 presents the estimated variance components and heritability for harvest body weight using PBLUP, GBLUP, and ssGBLUP models, with and without the inclusion of common environmental effects (i.e., full-sib group effects). When these effects were included, PBLUP yielded the highest heritability estimate (0.36 ± 0.22), followed by ssGBLUP (0.34 ± 0.06) and GBLUP (0.32 ± 0.05). The corresponding common environmental coefficients (*c*^2^) were 0.11 for PBLUP, 0.14 for GBLUP, and 0.13 for ssGBLUP. Excluding common environmental effects led to increased heritability estimates across all models, as the variance previously attributed to full-sib groups was reassigned to the additive genetic component.

As shown in Table 5, ssGBLUP achieved the highest prediction accuracy (0.80), representing a 6.67% improvement over PBLUP (0.75) and a 19.40% improvement over GBLUP (0.67). Prediction bias was also lowest for ssGBLUP (1.26), compared to PBLUP (2.04) and GBLUP (1.86). Notably, the lower prediction accuracy of GBLUP relative to PBLUP was unexpected and may demonstrate that under the current family structure and marker density, the genomic relationship matrix may have limited power to resolve within-family differences.

## 3. Discussion

Accurate pedigree information is essential for reliable EBV estimation, optimal mating decisions, and inbreeding control in family-based breeding programs [5,6,7]. In shrimp nucleus populations, traditional family tracing methods such as VIE tagging are widely used during communal rearing but are associated with tagging errors, size-dependent marking delays, and substantial environmental bias introduced during the early rearing stages [11,12,20]. To address these limitations, we advanced communal rearing to PL20 and implemented a molecular pedigree reconstruction strategy based on a 1K SNP panel containing 1125 markers. This approach eliminated the need for physical tagging, significantly improved the accuracy of family identification under commercial-scale conditions, and reduced early environmental confounding. Furthermore, the integration of genomic and pedigree data using the H matrix in the ssGBLUP model led to a clear improvement in the prediction accuracy of EBVs. Together, these results demonstrate that the 1K SNP panel is a practical and effective tool for supporting both pedigree assignment and genomic evaluation in *L. vannamei* breeding programs.

The number of SNPs required for accurate pedigree reconstruction depends on species, population structure, and marker informativeness. While some studies have shown that fewer than 100 SNPs may achieve over 95% parentage assignment in controlled settings [23,24,25,26], complete resolution of all individuals in large or genetically complex populations typically requires 300 to 500 SNPs with moderate to high MAF [7,20,27]. Several studies have further emphasized that marker informativeness, particularly minor allele frequency, strongly influences kinship assignment accuracy, as higher MAF contributes more discriminatory power per locus [22,28]. However, achieving 100% accuracy in individual identification within large populations is challenging without a sufficient number of markers, regardless of marker diversity. For example, fewer than 300 SNPs achieved 94–96% accuracy in *L. vannamei* and ~99% in *Penaeus monodon*, *Salmo salar* and *Larimichthys crocea*, but failed to fully distinguish all individuals [10,20,26,29,30,31]. In this study, the 1K SNP panel comprising 1125 loci enabled 100% full-sib family identification using COLONY, even though the tested population was not included in the original panel design. The high proportion of informative markers (87.7% with MAF > 0.30 and only 2.6% < 0.20, Figure 2) and their even genomic distribution contributed to this performance, supporting the panel’s suitability for pedigree reconstruction in large-scale breeding populations of *L. vannamei*.

In addition to demonstrating the panel’s assignment power, we established a practical workflow for pedigree reconstruction under commercial breeding conditions. Using parental genotypes and anchor individuals from the selection group, we first verified the accuracy of full-sib family clustering. The COLONY software (2.0.7.2) successfully distinguished 72 families with 100% consistency with recorded pedigree information. Notably, even when anchor and parental genotypes were excluded, sibship assignment using test individuals alone still recovered the same family structure, highlighting the robustness of the 1K SNP panel. However, anchor individuals remain essential for linking family IDs between the test and selection groups, especially when parental samples are partially missing, a common challenge in shrimp breeding due to physical loss of tags or tissue degradation. For instance, in our G5 generation, only 125 of 144 parents were available for genotyping. In such cases, we recommend genotyping one or more anchor individuals per family as a reliable backup strategy to ensure complete pedigree reconstruction. Despite previous reports that the use of excessive SNPs may lead to over-fragmentation of full-sib families or misclassification of unrelated individuals [7], this issue was not observed in our analysis. This may be attributed to the high quality and informativeness of the selected markers, as well as the well-defined family structure maintained through controlled mating in the nucleus population. These results confirm the reliability of this approach and its suitability for routine pedigree reconstruction in practical breeding scenarios.

Heritability estimates for harvest body weight ranged from 0.32 ± 0.05 to 0.36 ± 0.22 across the three models, with inclusion of common environmental (full-sib group) effects. These values are higher than those reported (0.17 ± 0.04 to 0.24 ± 0.05) in several previous studies on *L. vannamei*, indicating sustained additive genetic variance and potential for genetic improvement in this population [32,33]. As expected, accounting for full-sib group effects reduced heritability estimates by attributing part of the phenotypic variance to shared early rearing environments [34,35]. Although early communal rearing was advanced to PL20, the estimated common environmental effects still exist (Table 4). Under the present full-sib design, however, this effect should not be interpreted as a purely environmental effect, because it may reflect both shared environmental influences and dominance variance confounded within full-sib families. If a non-negligible fraction of this effect reflects dominance variance, the narrow-sense heritability may be underestimated. The relatively large standard error of the PBLUP estimate may be related to the family-based structure of the population, limited pedigree depth, and imbalance in family size, which reduce the resolution of pedigree-based relationships for partitioning additive genetic variance.

The 1K SNP panel also proved effective for genomic prediction of harvest body weight. Among the three methods, ssGBLUP achieved the highest prediction accuracy, with improvements of 6.67% over PBLUP and 19.40% over GBLUP. Similar findings have been reported across aquaculture species. For example, in *S. salar*, ssGBLUP improved prediction accuracy by 1.30% to 13.90% over PBLUP [36]; in *Macrobrachium rosenbergii*, the improvement reached 42.42% [37]; and in channel catfish, 28.00% [38]. These studies highlight the advantage of combining genomic and pedigree information in family-based breeding programs [39]. In our study, although GBLUP theoretically enables more precise estimation of additive genetic values by capturing Mendelian sampling, it performed worse than PBLUP. One possible explanation is that the 1K SNP panel provided insufficient marker density to accurately distinguish within-family differences in this full-sib-structured population, where each family included 13 to 30 individuals. Similar observations have been reported in other species, where GBLUP underperformed due to limited genomic resolution or small reference populations [40]. Notably, the prediction accuracy of ssGBLUP was also influenced by the relative weighting of genomic and pedigree information in the H matrix. When the G and A matrices were weighted at 0.95 and 0.05, respectively (default setting), ssGBLUP performed worse than PBLUP. However, adjusting the weights to 0.15 for G and 0.85 for A substantially improved prediction accuracy, indicating that greater reliance on pedigree relationships helped offset the limitations of genomic data under low marker density. These findings align with previous research showing that optimal weighting strategies in ssGBLUP can enhance prediction performance in family-structured populations with sparse genotyping [41]. This highlights the importance of tuning the H matrix when implementing ssGBLUP under varying genotyping conditions. It should also be noted that the corrected phenotype used in this study was defined as the observed phenotype after removing fixed effects, and therefore retained additive genetic, common environmental, and residual components. Under such a definition, prediction accuracy should be interpreted with caution, especially in a dataset with a pronounced family structure, because models that are better able to capture family means or other sources of shared family-level variation may show correspondingly higher correlations with the validation target. In addition, prediction bias values were greater than 1.0 for all models. This pattern may be related to several factors, including limited information in the reference population, the pronounced family structure of the population, and relatively limited marker density.

In addition to pedigree reconstruction and EBV prediction, the panel supported genetic structure and diversity analyses. PCA, Admixture analysis, and neighbor-joining trees clearly separated founder populations, and diversity metrics such as He, Ho, and Pi remained stable across generations, indicating that the breeding population maintained a broad genetic base. These results support the broader applicability of the panel for population monitoring and genetic management. Moreover, the liquid SNP panel platform is cost-effective and scalable for large-scale breeding operations. In our study, nearly 2000 individuals were genotyped within two weeks at a cost of approximately 40 RMB (approximately 5.5 USD, under a standard fee-for-service arrangement) per sample, including DNA extraction, library construction, and sequencing, with a per-SNP cost of $0.005 per individual. The panel provides a practical and affordable solution for routine use in commercial *L. vannamei* breeding programs. Future work may explore further reductions in cost, integration of trait-specific markers, and validation of panel performance in mass-spawning populations.

## 4. Materials and Methods

### 4.1. Design of 1K SNP Panel

The 1K SNP panel used in this study is part of the “Yellow Sea Array No.1”, a multi-density SNP array system designed to support genomic selection and pedigree reconstruction in *L. vannamei*. It was derived from the 10K panel, which in turn was developed based on the 55K panel constructed using whole-genome resequencing (WGS) data (Reference genome: ASM378908V1). The design of the 55K panel involved WGS of 433 individuals from eight genetically diverse populations, including commercial lines and wild populations from Ecuador. The 55K panel was constructed through a two-stage process, integrating quality-filtered SNPs (e.g., Minor Allele Frequency (MAF) > 0.05, call rate > 0.90, heterozygosity > 0.10) with functional markers. This resulted in a final set of 56,214 SNPs optimized for genome-wide coverage and genotyping accuracy. The 10K panel was subsequently derived by selecting 10,416 informative and evenly distributed SNPs from the 55K panel, using genotyping data from 2330 individuals and optimized via the MOLO algorithm [42]. These two panels formed the technical foundation for the 1K SNP panel developed in this study, with full details provided in a separate manuscript (in preparation).

To construct the 1K panel, we used the same dataset of 2330 individuals genotyped with the 55K panel. After standard quality control using PLINK 1.9 [43], SNPs with heterozygosity values between 0.20 and 0.60, MAF > 0.05, and call rate > 0.90 were retained. The MOLO algorithm was applied to select 1125 SNPs by maximizing locus-average Shannon entropy ($\bar{H_{L}}$) while ensuring even genomic distribution and inclusion of mandatory loci. The weights for MAF-based informativeness (w1) and genomic uniformity (w2) were both set to 0.5, reflecting equal prioritization of marker information content and genome-wide coverage. Mandatory loci were defined as: (i) SNPs located at the start and end positions of each linkage group to ensure full chromosomal representation, and (ii) loci previously associated with economically important traits based on prior studies. The average practical call rate of these loci in 2330 individuals was 0.99. Probes were designed using the GenoBaits platform (MolBreeding, Beijing, China). All selected SNPs were successfully incorporated into the final 1K liquid-phase SNP panel, and specific chemical modifications were incorporated into the probes to reduce non-specific interactions while preserving their binding affinity to the target genomic regions.

### 4.2. Nucleus Population Construction and Early-Stage Communal Rearing Design

The Nucleus Population (NP) used in this study was established at Zhongzheng Aquatic Science and Technology Co., Ltd. (Dongfang, Hainan Province, China), and it has undergone five successive generations: G0 and G1 in 2020, G2 in 2021, G3 in 2022, and G4 in 2023. This selective breeding program employed a family-based selection strategy, with a primary focus on improving growth traits. A mean generation interval of approximately 9 months was maintained, and an average of more than 70 families were constructed per generation. In generations G0 through G4 (2020–2023), performance testing was conducted through traditional full-sib family comparison. Individuals in the test group were tagged using VIE tags and communally reared, while individuals in the selection group were maintained in separate family tanks to preserve pedigree integrity.

In the G5 generation, the initial communal rearing time was advanced to the early post-larval (PL20) stage, and family identification was conducted using the 1K SNP panel instead of VIE. Broodstock from the G4 generation reached sexual maturity in September 2023, and pair mating was conducted in November 2023 based on EBVs and inbreeding control within the NP. Fertilized females were transferred to separate 1 m^3^ tanks for spawning and incubation, and hatched nauplii were reared in isolated 500 L tanks. Parent prawns were marked with specific eyestalk rings after artificial insemination and spawning. To minimize inter-family variation in hatching date, no mating design for half-sib families was outlined. A total of 81 full-sib families were produced within a 14-day controlled mating window. Larval standardization was conducted twice: 20,000 nauplii were selected at the N3–N4 stage, and 3000 post-larvae at the PL5 stage. At PL20, 72 families that met pathogen-free and survival rate criteria were retained for subsequent breeding. In the selection group, 600 individuals per family were stocked into independent 3 m^3^ biosecurity tanks, with strict inter-family separation to prevent cross-contamination. These candidate broodstock would be selected for future mating based on EBVs derived from family-level performance data. To evaluate growth performance, the remaining individuals were assigned to a test group. To ensure equal family representation and reduce environmental variance, each family was first reared separately for 34 days in isolated larval tanks.

Subsequently, three replicate groups of 30 post-larvae per family were randomly selected and communally reared in separate indoor concrete tanks (8 m^2^ surface area; 0.8 m water depth) under controlled conditions. Initial body length was recorded for each family prior to communal stocking. The stocking density was approximately 337 individuals/m^3^. Shrimp were fed five times daily at 5–10% of body weight, with a daily water exchange of 20–50% depending on water quality. The water temperature was maintained between 27 °C and 30 °C throughout the trial.

### 4.3. Sampling and Genotyping

After 100 days of growth, one test tank with superior performance was selected for sampling. All surviving individuals were collected and assigned unique IDs, and their sex, body weight, and body length were recorded individually. Muscle tissue was dissected from each shrimp and preserved in 95% ethanol. Samples were also collected from the parents of the G5 families. To support family identification using the 1K SNP panel, some individuals from each family in the selection group were randomly sampled and designated as anchor individuals (i.e., reference individuals with known family IDs). These individuals served as reference genotypes during COLONY analysis, enabling accurate assignment of test group clusters to specific families, especially when parental genotypes were missing or incomplete. The number of anchor individuals selected depended on parental sample availability: one anchor if both parents were available, two if only one parent was available, and four if neither parent was available. In total, 1818 individuals were sampled from the test group, along with 117 parents (56 paternal and 61 maternal, 27 were excluded due to mortality or loss of eyestalk rings) of the G5 families and 103 anchor individuals from the selection group.

Genotyping was performed at MolBreeding Biotechnology Co., Ltd. (Shijiazhuang, Hebei, China) using the 1K SNP panel. High-quality DNA was extracted from muscle tissue using the GenoPrep Polyphenol DNA Extraction Kit (magnetic bead-based method). DNA quality and concentration were assessed with the DropAnalyzer Plus workstation (HC Scientific Technology Co., Ltd., Beijing, China), which evaluates nucleic acid purity and yield. Targeted sequencing libraries were prepared using GenoBaits probe capture technology, and sequencing was conducted on the DNBSEQ-T7 platform (MGI Tech Co., Ltd., Shenzhen, China). SNP quality control was performed using PLINK 1.9 [43]. SNPs with MAF < 0.05 or call rate < 0.90 were removed, and individuals with a missing genotype rate > 0.20 were excluded. MAF and polymorphism information content (PIC) were calculated for each analyzed population to assess genetic diversity and marker informativeness.

### 4.4. Population Genetic Structure and Genetic Diversity Analysis

As part of the initial founder population, three representative batches of commercial fast-growth lines introduced from the CP Group were designated as C1, C2, and C3. Each batch comprised approximately 10 full-sib families and was independently introduced during the early stage of the breeding program. To assess the suitability of the 1K SNP panel for population genetic analysis, a total of 46 archived samples from these three batches were genotyped, including 19 individuals from C1, 15 from C2, and 12 from C3. Principal Component Analysis (PCA) was performed using PLINK and visualized with the R package ggplot2 V4.0.2. Population structure was analyzed using Admixture 1.3 with default parameters, testing K values ranging from 1 to 5. The K value with the minimum cross-validation error (CV error) was chosen as the best-fit number of clusters. Admixture output was visualized using the R package pophelper V2.3.1 [44]. A neighbor-joining phylogenetic tree was constructed using PHYLIP [45], and an Identity by State (IBS) genetic distance matrix was constructed based on SNPs and bootstrap analysis with 1000 replications. The phylogenetic tree was visualized with the R package ggtree V4.2.0 [46]. Genetic diversity parameters, including observed heterozygosity (Ho), expected heterozygosity (He), PIC, and nucleotide diversity (Pi), were calculated using PLINK and VCFtools [47]. Analyses were performed separately for three groups: the founder population (46 samples), the G5 test group (1818 samples), and the G5 parents (117 samples).

### 4.5. Parentage and Sibship Assignment

Parentage and sibship assignment were performed using COLONY v2.0.7.0 [48], applying the full-likelihood method under the assumptions of monogamy for both sexes, no inbreeding, and a weak sibship prior. A 95% confidence threshold was used for all assignments. In the first step, only anchor individuals were used to establish family clusters. COLONY assigned these anchors into distinct sibships and generated virtual sires and dams for each family. The accuracy of this clustering was evaluated by comparing the inferred family assignments with known family records, which also served as the baseline for subsequent test individual assignment. In the second step, parental genotype data were incorporated into the COLONY analysis. Whenever available, real parents replaced the previously assigned virtual sires and dams. To ensure reliable integration of real parental genotypes, consistency between COLONY assignments and original parental records was checked. This step served to validate and update family structure with higher confidence, particularly for families with complete or partial parental information. In the third step, all test individuals were assigned to the established families by COLONY, using the known anchor individuals and real parents as references. Assignments were accepted as accurate based on the validated structure from previous steps. This approach enabled comprehensive pedigree reconstruction, even for families lacking parental samples, by relying on well-identified anchor individuals.

To assess the robustness of the 1K SNP panel for sibship inference, we performed an independent COLONY run using only the genotypes of the 1818 test individuals—excluding both anchor individuals and parental data. The resulting sibship clustering was then compared to the full-reference approach to evaluate consistency and assignment stability under minimal prior information. To visually summarize the family-level genetic structure, a phylogenetic heatmap depicting pairwise relationships between inferred families was generated using the R package ComplexHeatmap V2.26.1.

### 4.6. Estimation of Genetic Parameter and Prediction Accuracy of EBVs

Variance components for harvest body weight were estimated using ASReml software V4.2 [49] under three animal models: pedigree-based best linear unbiased prediction (PBLUP), genomic BLUP (GBLUP) and single-step genomic BLUP (ssGBLUP). These models utilized relationship matrices A, G and H, respectively. The A matrix was constructed from recorded pedigree information of 14,671 individuals from G0 to G5 with kinship coefficients ranging from 0 to 0.75. The G matrix was derived from G5 SNP genotypes following the method of VanRaden [50] with kinship coefficients ranging from −0.033 to 0.15. The H matrix was constructed with G and A matrices [51] blended at a ratio of 0.15: 0.85 (the results of the sensitivity analysis, conducted across a range of blending weights, are shown in Supplementary Figure S4), resulting in kinship coefficients ranging from −0.028 to 0.78. To evaluate the impact of common environmental effects, animal models were fitted with and without the inclusion of the common environmental effect, which accounts for both shared environment and one-quarter of the dominance variance within full-sib families. The full model was as follows:

(1) $$y_{ijk}=\mu +{Sex}_{i}+{\beta BL}_{j}\left({Sex}_{i}\right)+a_{j}+c_{k}+e_{ijk}$$

where $y_{ijk}$ is the observed harvest body weight of the *j*-th individual; μ is the overall mean; ${Sex}_{i}$ is the fixed effect of the *i*-th sex; ${BL}_{j}\left({Sex}_{i}\right)$ is a linear covariate of the initial body length of the *j*-th individual nested within the *i*-th sex, β is the regression coefficient of the covariate; $a_{j}$ is the additive genetic effect of the *j*-th individual assumed to be normally distributed, $a_{j}\sim \left(0,{M\sigma }_{a}^{2}\right)$, where *M* is the relationship matrix among individuals (A, G, or H), $\sigma_{a}^{2}$ is the additive genetic variance; $c_{k}$ is the group effect for the *k*th full-sib family, which is due to separate rearing of the full-sib families before communal rearing and one-quarter of the non-additive genetic effect (dominance) common to full-sibs, $c_{k}\sim (0,{I\sigma }_{c}^{2})$, *I* is the identity matrix, $\sigma_{c}^{2}$ is the variance of the common environmental effect (primarily full-sib effect); and $e_{ijk}$ is the random residual error of the *j*-th individual, $e\sim (0,{I\sigma }_{e}^{2})$, where $\sigma_{e}^{2}$ is the residual variance. When the model does not include the common environmental effect, $c_{k}$ is removed. All fixed effects in the model were statistically significant based on the Wald test in ASREML. The phenotypic variance $\left(\sigma_{p}^{2}\right)$ was calculated as $\sigma_{p}^{2}=\;\sigma_{a}^{2}+\sigma_{c}^{2}+\;\sigma_{e}^{2}$. When the model does not include the common environmental effect, $\left(\sigma_{p}^{2}\right)$ was calculated as $\sigma_{p}^{2}=\;\sigma_{a}^{2}+\;\sigma_{e}^{2}$. The narrow-sense heritability was estimated as $h^{2}=\sigma_{a}^{2}/\sigma_{p}^{2}$; the common environmental coefficient is calculated as $c^{2}=\;\sigma_{c}^{2}/\sigma_{p}^{2}$.

To evaluate the predictive performance of each model, a 5-fold cross-validation was conducted. The dataset was randomly divided into five equal, mutually exclusive subsets. In each iteration, one subset was designated as the validation set (with phenotypes masked), and the remaining four were used for model training. EBVs or GEBVs for individuals in the validation set were predicted using the trained models (PBLUP, GBLUP, and ssGBLUP) as described above.

The prediction accuracy was calculated as the Pearson correlation between the predicted values and the corrected phenotypes, divided by the square root of heritability estimated from the PBLUP model, which was used as a common denominator across all models to ensure direct comparability:

(2) $$r_{\left(GEBV\;or\;EBV,\;y\right)}=\frac{cor\;\left(GEBV\;or\;EBV,y\right)}{h}$$

The corrected phenotype was computed as the sum of EBV, common environmental effect and residual. The prediction bias was estimated as the regression coefficient of corrected phenotypic values and predicted GEBVs (EBVs). To ensure robustness, the entire cross-validation process was repeated 10 times, and the final accuracy and bias estimates were averaged across all folds and repetitions.

## 5. Conclusions

This study demonstrates the development and validation of a cost-effective 1K SNP panel as part of the “Yellow Sea Array No.1” for pedigree reconstruction and genomic evaluation in *Litopenaeus vannamei* breeding programs. The panel enabled accurate family identification under early communal rearing conditions and facilitated the establishment of a practical SNP-based pedigree framework for large-scale selection. When integrated into genetic evaluation, it improved the prediction accuracy of breeding values through the ssGBLUP method, particularly when pedigree and genomic information were optimally integrated, highlighting the advantage of combining both sources of relationship information. In addition to trait evaluation, the panel also proved effective for population structure analysis and genetic diversity monitoring, showing consistent performance across generations. Together, these results support the utility of this SNP panel as a versatile and scalable tool for enhancing the efficiency and precision of selective breeding in commercial shrimp production.

## Figures and Tables

**Table 1:** MAF and PIC statistics in three groups.

Group	Parameter	Number	Minimum	Maximum	Mean	Standard Deviation
Parent	MAF	1081	0.06	0.50	0.40	0.08
	PIC	1081	0.10	0.38	0.36	0.03
Anchor	MAF	1092	0.05	0.50	0.39	0.08
	PIC	1092	0.10	0.38	0.36	0.04
Test	MAF	1082	0.05	0.50	0.40	0.08
	PIC	1082	0.10	0.38	0.36	0.03

**Table 2:** PIC, Pi, observed heterozygosity (Ho) and expected heterozygosity (He) across three generations based on 1125 SNPs from the 1K panel.

Population	PIC	Pi	Ho	He
Subset of founders	0.35	0.46	0.45	0.45
Parents of G5	0.36	0.47	0.46	0.47
G5	0.36	0.47	0.46	0.47

**Table 3:** Descriptive statistics of harvest body weight by sex.

Group	Number	Min (g)	Max (g)	Mean (g)	SD (g)	CV (%)
Female	890	1.50	33.10	18.01	4.58	25.41
Male	928	3.80	32.00	17.51	4.46	25.46
Total	1818	1.50	33.10	17.75	4.52	25.47

**Table 4:** The variance components and heritability estimates for harvest body weight.

Method	$\sigma_{p}^{2}$	$\sigma_{a}^{2}$	$\sigma_{c}^{2}$	$\sigma_{e}^{2}$	$h^{2}$	$c^{2}$
PBLUP	20.32 ± 1.33	12.91 ± 2.53	/	7.41 ± 1.36	0.64 ± 0.09	/
	19.47 ± 1.26	7.04 ± 4.45	2.09 ± 1.71	10.34 ± 2.28	0.36 ± 0.22	0.11 ± 0.09
GBLUP	18.51 ± 0.81	8.73 ± 1.05	/	9.78 ± 0.58	0.47 ± 0.04	/
	19.43 ± 1.06	6.29 ± 1.13	2.68 ± 0.93	10.45 ± 0.62	0.32 ± 0.05	0.14 ± 0.04
ssGBLUP	18.85 ± 0.85	9.19 ± 1.11	/	9.66 ± 0.59	0.49 ± 0.04	/
	19.64 ± 1.08	6.73 ± 1.20	2.56 ± 0.93	10.35 ± 0.63	0.34 ± 0.06	0.13 ± 0.04

$\sigma_{p}^{2}$: phenotypic variance; $\sigma_{a}^{2}$: additive genetic variance; $\sigma_{c}^{2}$: full-sib effect variance; $\sigma_{e}^{2}$: residual variance; $h^{2}$: heritability; $c^{2}$: common environmental coefficient.

**Table 5:** Predictive accuracy of the EBV/GEBV for harvest body weight with three methods.

Method	Predictive Accuracy	Bias
PBLUP	0.75	2.04
GBLUP	0.67	1.86
ssGBLUP	0.80	1.26

Bias: the regression coefficient of corrected phenotypic values and predicted GEBVs (EBVs).

## Data Availability

The data presented in this study are available on request from the corresponding author due to the protection of breeding population information.

## Supplementary Materials

The following supporting information can be downloaded at: https://www.mdpi.com/article/10.3390/ijms27114665/s1.
