# Supplementary material for: Development and Applications of a 1K SNP Panel for Whiteleg Shrimp: From Pedigree Reconstruction to Genomic Selection
Source: Int J Mol Sci. 2026 May 22;27(11):4665. doi: 10.3390/ijms27114665 (PMC13257326; doi:10.3390/ijms27114665)
Supplement: Supplementary file 1 [file ijms-27-04665-s001.zip › ijms-4268026-supplementary.pdf]

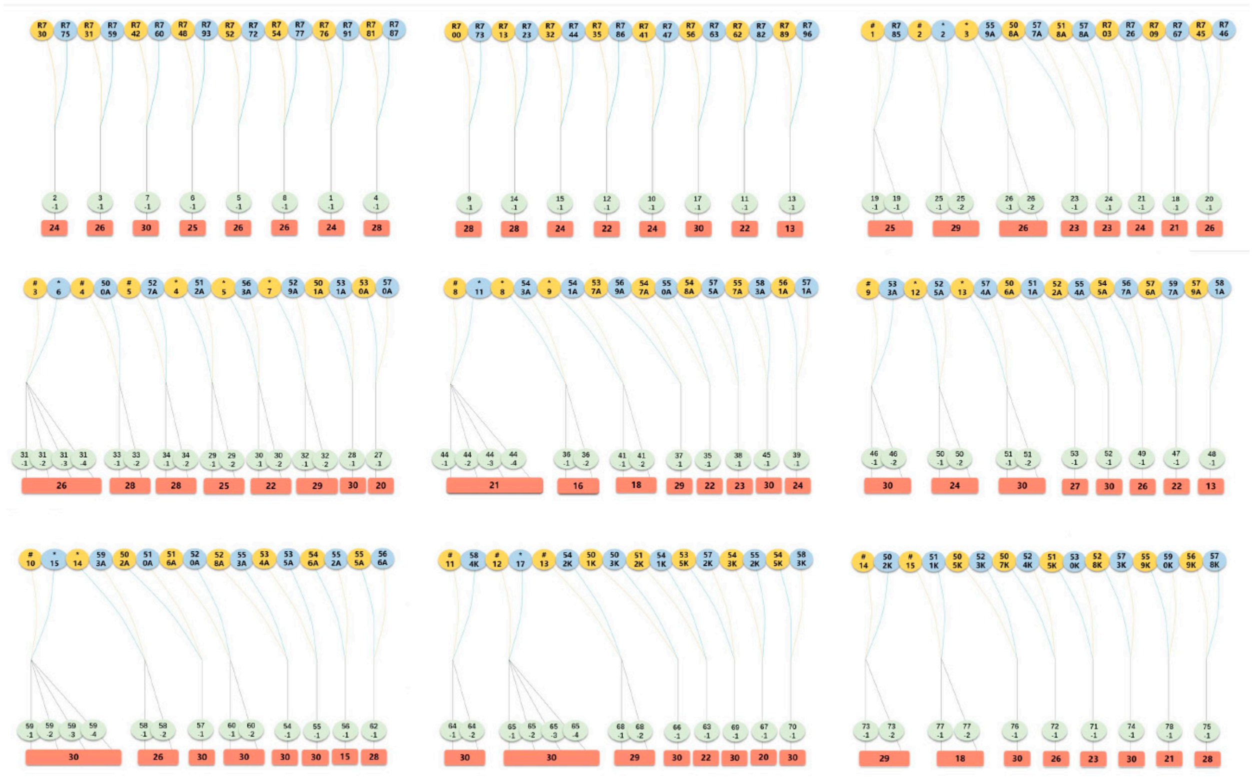

Supplementary Figure S1. Detailed pedigree results of 72 families identified by Colony. The upper section shows the virtual parents (denoted by * and #) or real parents (eyestalk ring IDs), while the lower section presents the family IDs of the anchor individual (green box) and the number of assigned test individuals (red box).

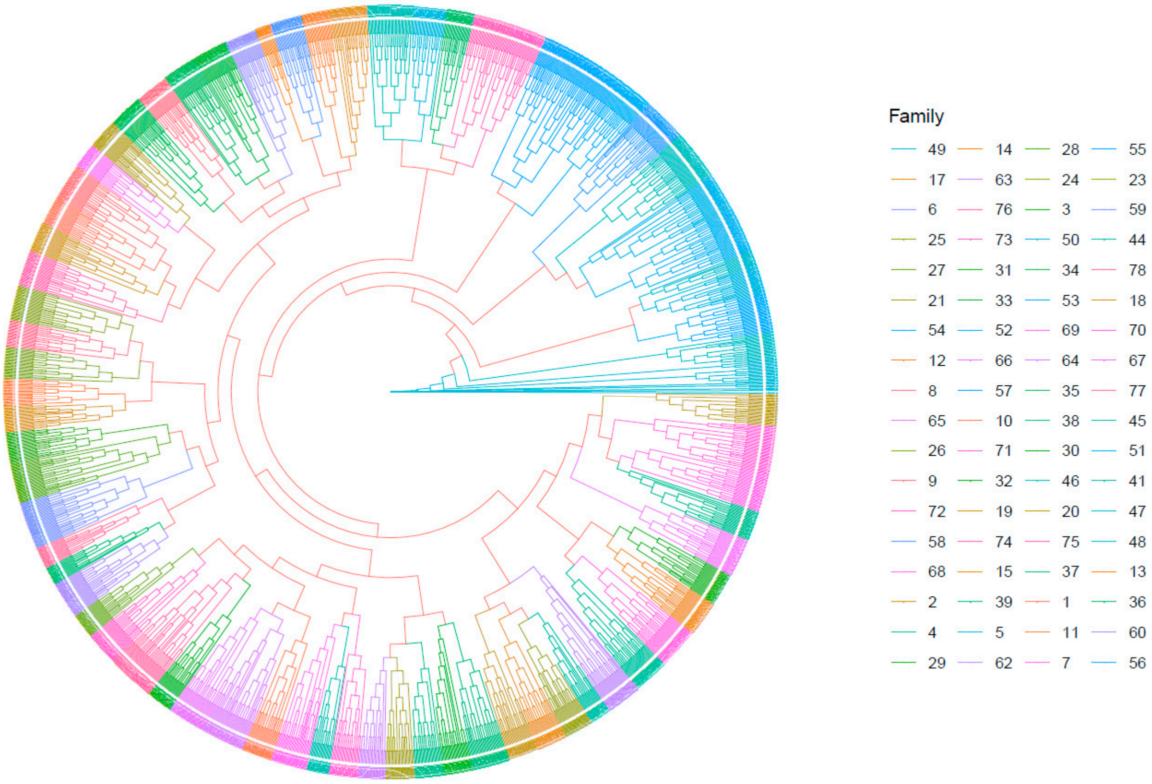

Supplementary Figure S2. Phylogenetic tree of 1,818 test individuals from 72 families. Different colors represent individuals from different families.

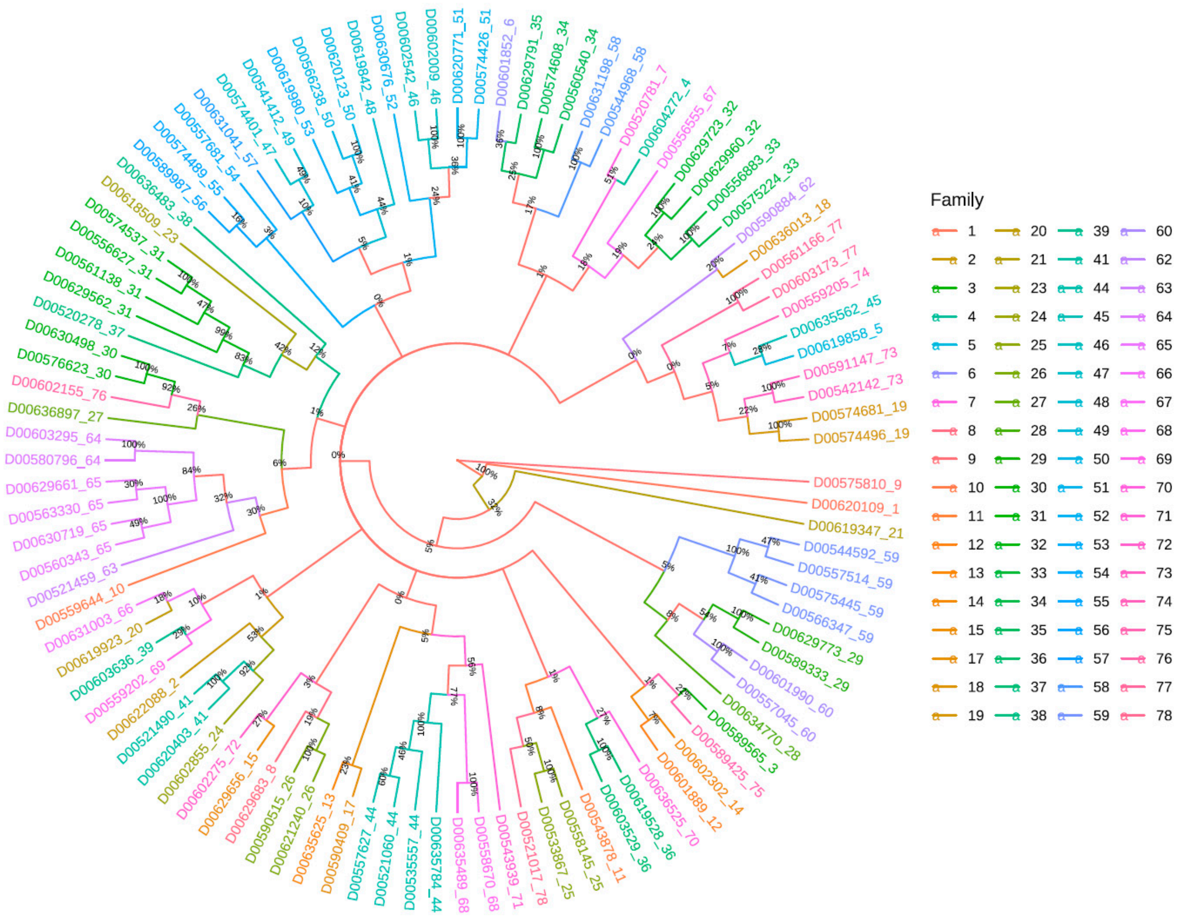

Supplementary Fig. S3 Phylogenetic tree of 103 anchor individuals with bootstrap support values.

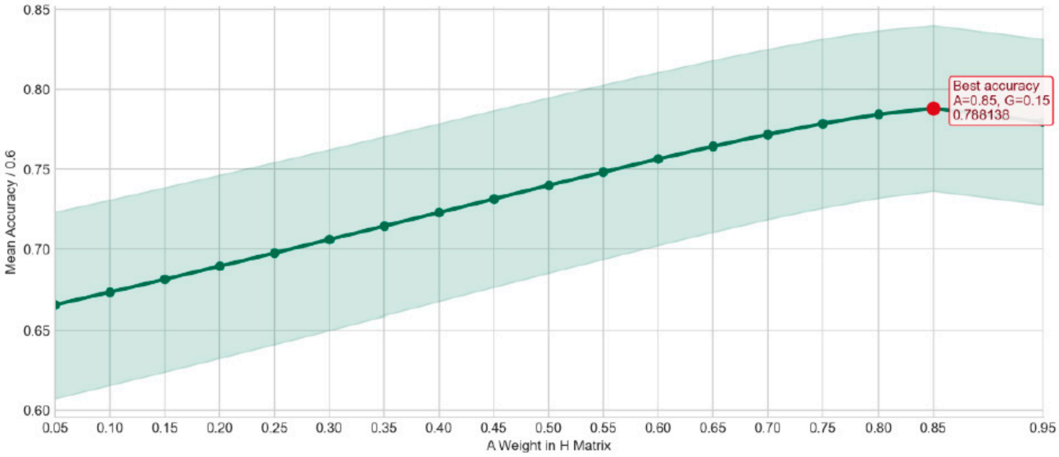

(A)

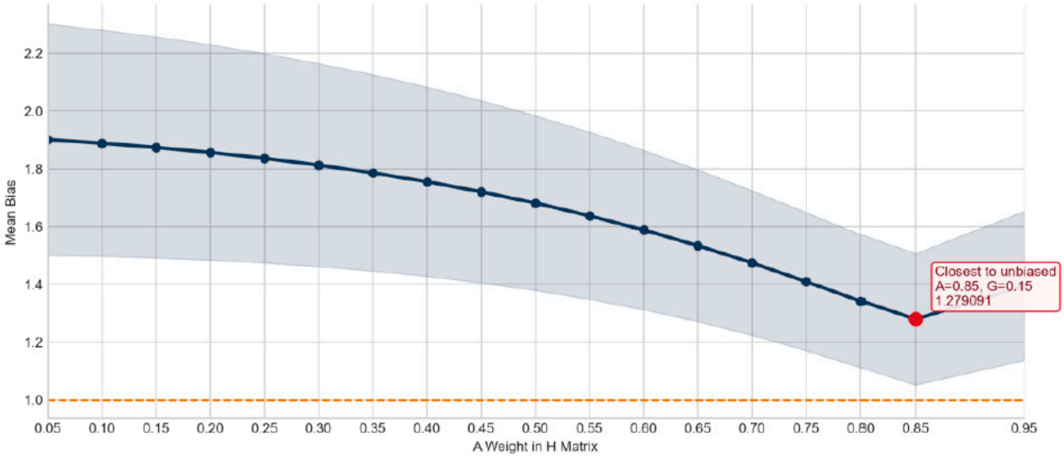

(B)

Supplementary Figure S4. Effect of G/A blending weights on predictive accuracy (A) and predictive bias (B) in ssGBLUP.
